# Inflammatory marker profiles vary by BMI across Crohn’s disease, ulcerative colitis, psoriasis, and psoriatic arthritis

**DOI:** 10.3389/fendo.2026.1851905

**Published:** 2026-07-17

**Authors:** Stephen A. Shields, Ann Von Holle, Ruthvik Padival, Joseph F. Merola, Rachael Zuckerman, Jeff T. Mohl, Elizabeth L. Ciemins

**Affiliations:** 1 Research & Analytics Department, AMGA (American Medical Group Association), Alexandria, VA, United States; 2 Intermountain Health, Gastroenterology and Digestive Health, Salt Lake City, UT, United States; 3 Department of Dermatology, University of Texas Southwestern Medical Center, Dallas, TX, United States

**Keywords:** body mass index, Crohn’s disease, immune-mediated inflammatory disease, inflammatory biomarker, obesity, psoriasis, psoriatic arthritis, ulcerative colitis

## Abstract

**Background:**

Obesity represents a state of chronic low-grade inflammation, yet comprehensive comparative analyses examining systemic inflammatory markers across multiple immune-mediated inflammatory diseases (IMIDs) in relation to body mass index (BMI) categories remain limited. We evaluated how obesity-associated inflammatory burden manifests longitudinally across immunologically distinct conditions.

**Methods:**

We conducted a longitudinal cohort study using de-identified electronic health record data from the Optum Labs Data Warehouse, selecting adult patients with Crohn’s disease (CD), ulcerative colitis (UC), psoriasis (PsO), or psoriatic arthritis (PsA) with at least two BMI measurements over five years. Using generalized mixed effects regression models, we estimated associations between BMI categories (healthy weight, <25 kg/m²; overweight, 25.0–29.9 kg/m²; obesity, ≥30 kg/m²) and C-reactive protein (CRP), erythrocyte sedimentation rate (ESR), and ferritin. We compared patients with obesity and patients with overweight plus a high-risk comorbidity to the healthy-weight referent.

**Results:**

Across all four conditions, patients with obesity demonstrated significantly elevated CRP (49–83% higher) and ESR (24–48% higher) at baseline compared to healthy-weight counterparts. Patients with overweight plus high-risk comorbidities showed intermediate elevations (CRP 34–55% higher, ESR 13–30% higher). These patterns attenuated over time but remained statistically significant through year 5 in nearly all cohorts. Ferritin demonstrated near null associations across all conditions.

**Conclusion:**

Consistent CRP and ESR elevations across four immunologically distinct IMIDs, combined with null ferritin associations, suggest that excess adiposity may contribute to systemic inflammatory burden regardless of underlying disease mechanism, warranting further investigation of weight optimization as part of comprehensive IMID management.

## Introduction

Obesity is now recognized as a state of chronic low-grade inflammation, with adipose tissue functioning as an active source of inflammation (1, 2). This systemic inflammation is expected to manifest in elevated acute-phase reactants, a class of proteins that include C-reactive protein (CRP) and erythrocyte sedimentation rate (ESR), and ferritin, which are routinely measured in clinical practice to assess inflammatory disease activity (3, 4). These three markers differ in their immunological characteristics. CRP is synthesized by hepatocytes primarily in response to IL-6 stimulation, serving as a sensitive marker of systemic inflammation (5). ESR reflects plasma protein concentrations including fibrinogen and immunoglobulins, providing a non-specific measure of inflammatory activity (6). Ferritin functions both as an acute-phase reactant and iron storage protein, with dual regulation by inflammation and iron metabolism (7, 8).

Immune-mediated inflammatory diseases (IMIDs), including Crohn’s disease (CD), ulcerative colitis (UC), psoriasis (PsO), and psoriatic arthritis (PsA), affect millions of individuals worldwide (9–11). Crohn’s disease and ulcerative colitis, collectively known as inflammatory bowel disease (IBD), involve chronic intestinal inflammation and may include extraintestinal inflammatory manifestations (9). Psoriasis presents as chronic inflammatory skin lesions, while psoriatic arthritis combines skin and joint inflammation (12, 13). Despite their different clinical presentations and tissue targets, these conditions share common features of chronic immune system dysregulation requiring ongoing monitoring and management of inflammatory activity.

The intersection of obesity and IMIDs has important clinical implications. Epidemiological evidence shows that elevated body mass index (BMI) increases the risk of developing psoriasis and psoriatic arthritis (14, 15). In IBD, obesity is associated with more complicated disease courses, increased hospitalization rates, and poorer surgical outcomes (16). Patients with obesity and IMIDs also demonstrate diminished response to biologic therapies and experience higher rates of cardiovascular and metabolic comorbidities (17, 18). These observations raise questions about whether obesity represents a comorbid condition or whether it fundamentally alters the inflammatory profile of patients with IMIDs.

Current evidence regarding inflammatory markers in patients with both obesity and IMIDs comes primarily from single-disease studies or cross-sectional analyses. Studies in psoriasis have documented significantly elevated CRP levels in patients with higher BMI (19), while IBD research has shown similar patterns (20). What remains unclear is whether inflammatory markers follow a consistent pattern across immunologically distinct diseases and across BMI categories, or whether these markers are differentially affected depending on the disease. To our knowledge, such studies have not been undertaken.

We conducted a multi-disease study examining CRP, ESR, and ferritin trajectories over five years in 17,710 patients with CD, UC, PsO, or PsA. Given that obesity is associated with elevated pro-inflammatory cytokines and that CRP, ESR, and ferritin are all classified as acute-phase reactants, we hypothesized that patients with higher BMI would demonstrate elevated levels across all three markers compared to patients with healthy weight, and that these elevations would be consistent across immunologically distinct diseases. Because these markers differ in their regulatory pathways and biological functions, examining all three provides an opportunity to characterize whether obesity-associated inflammation manifests as broad inflammatory activation or whether certain markers are more sensitive to adiposity-driven changes than others. Furthermore, if adipose tissue is a persistent inflammatory source, we would expect inflammatory marker elevations to be sustained over a multi-year follow-up period rather than resolving over time.

## Methods

### Study design and data source

We conducted a longitudinal retrospective cohort study using data from the Optum Labs Data Warehouse (OLDW), a de-identified, national, longitudinal, real-world data asset containing clinical and administrative data from approximately 350 million patient lives. The OLDW integrates electronic health record (EHR) data from more than 50 healthcare organizations across the United States and contains demographic information, diagnoses, laboratory results, vital signs, and medication utilization in real-world clinical settings.

The study period extended from January 1, 2019, to June 30, 2024, with an index year of 2019. The index date was defined as the date of the patient’s first qualifying visit between 1/1/2019 and 12/31/2019. Qualifying visits were defined as any evaluation and management visit with any of the following providers: internal medicine, family medicine, general medicine, geriatrics, gynecologist, dermatologist, rheumatologist, gastroenterologist, or immunologist/allergist. Follow-up visits were counted yearly and analyzed at years 1, 3, and 5 post index date (± 60 days). In cases of multiple visits in this window per patient per follow-up year, the visit closest to the index date plus 1, 3, or 5 years was used. The study protocol was submitted to Advarra IRB and determined to be exempt from IRB oversight under 45 CFR 46.104(d)(4).

### Study population

Adults aged 18–79 years were included if they had at least one qualifying visit with a primary care provider or specialist during the 2019 index year, along with at least one prior visit 6–24 months earlier.

Patients were required to have at least two documented diagnoses of the same IMID (CD, UC, PsO, or PsA) occurring more than 30 days apart and within 730 days prior to the index date. Patients with multiple IMIDs were included in each disease-specific analysis for which they met the inclusion criteria. A patient with more than one qualifying IMID could contribute to more than one disease cohort. Because the cohorts were analyzed separately rather than pooled, this overlap does not introduce within-model duplication, but the disease-specific cohorts are not mutually exclusive. Disease identification used ICD-9 and ICD-10 diagnostic codes (**Supplementary Table 1**).

Patients were also required to have at least two BMI measurements: one within 60 days of the index date and one within the one-year follow-up window (i.e., index date + 1 year, ± 60 days). Patients were also required to have documented activity in the health system at each subsequent annual follow-up window (i.e., index date + n years, ± 60 days).

Patients were excluded if they had evidence of death, hospice care, or palliative care within one year following the index date. Pregnancy within one year post-index resulted in exclusion due to expected BMI effects. Patients with BMI <18.5 kg/m² (underweight) at index were excluded. Death, hospice, palliative care, or pregnancy occurring during follow-up were treated as censoring events.

### Exposure and outcomes

BMI was calculated as weight in kilograms divided by height in meters squared (kg/m²) using the most recent measurement within 60 days of each time point (i.e., index + n years, ± 60 days). We classified BMI according to criteria set by the Centers for Disease Control and Prevention: normal or healthy weight (HW, 18.5-24.9 kg/m², reference category), overweight (25.0-29.9 kg/m²), and obesity (≥30.0 kg/m²) (21). We additionally stratified overweight patients by the presence of high-risk conditions of cardiovascular disease, hypertension, diabetes, and dyslipidemia. This stratification aligns with treatment eligibility criteria for pharmacologic weight management, specifically glucagon-like peptide-1 (GLP-1) receptor agonists, which are indicated for patients with BMI ≥27 kg/m² in the presence of weight-related comorbidities (22). This stratification was chosen so that analyses were conducted on a clinically actionable population. Biologically implausible BMI values (<10 or >100 kg/m²) were excluded, though none were identified.

Primary outcomes were three inflammatory markers; CRP, ESR, and ferritin (5, 6, 8). Though ferritin is most relevant for CD and UC, we also assessed ferritin levels among patients with PsO and PsA for consistency.

Laboratory values were extracted from EHR results and expressed in mg/L for CRP, in mm/hr for ESR, and in ng/mL for ferritin. Inflammatory markers were captured on the index date within 60 days and at each follow-up time point post-index, ± 60 days. Biologically implausible values (CRP <0 or >500 mg/L; ESR <0 or >150 mm/hr; ferritin <0 or >5,000 ng/mL) were excluded. When multiple measurements occurred within the ±60-day window, the value closest to the target anniversary date was selected.

### Statistical analysis

Characteristics of the study population were assessed at baseline to identify potential differences across BMI categories. Continuous variables (i.e., age, BMI, inflammatory markers) were summarized using means, standard deviations, medians, and interquartile ranges when appropriate. Categorical variables (e.g., gender, race, ethnicity, insurance type) were reported as frequencies and percentages and tested using Chi-square tests or Fisher’s exact tests. Comparisons of continuous variables across the three BMI cohorts used one-way ANOVA or Kruskal-Wallis tests.

To examine whether BMI category was associated with differential inflammatory marker levels over time, associations between BMI categories and inflammatory markers over time were analyzed using generalized linear mixed-effects models (GLMM) with random intercepts for individual patients and a gamma distribution and log link. Product terms between BMI category and time as continuous variables were used to estimate differential trajectories. The outcome of interest was the ratio of means comparing each BMI category to the HW reference group, with ratios greater than 1.0 indicating elevation compared to the reference group and ratios less than 1.0 indicating reduction compared to the reference group. Ratios of means are expressed as percent higher than or lower than patients with HW.

To minimize confounding and isolate the independent association of BMI with inflammatory markers, models adjusted for potential confounders and included inverse probability weighting (IPW) using propensity scores. Baseline covariates included age at index, gender, race/ethnicity, and Elixhauser comorbidity score to capture patient comorbidity burden (dichotomized as 0 vs. >0). Time-varying covariates captured BMI changes, biologic medication use, and corticosteroid use at each assessment. For UC and CD cohorts, models additionally adjusted for 5-aminosalicylic acid (5-ASA) use, given its established anti-inflammatory properties and predominant use in IBD, which could independently influence marker levels. To address time-varying confounding affected by prior exposure, we employed a marginal structural model approach (23) with IPW using propensity scores generated with the ipw R package (24). We considered the categorical BMI variable as the exposure, and the weights were estimated using a multinomial distribution with a logit link. Weights were stabilized with baseline age and gender variables. Time-varying weights were updated annually across the five-year observation period to reflect changes in BMI category and time-varying covariates at each assessment. Propensity score models included baseline demographics, comorbidities, and time-varying medications, with stabilized weights calculated using baseline characteristics.

In estimating associations with the mixed-effects models using full likelihood methods mentioned above and assuming data were missing at random, we assume that the estimates are unbiased (25) without requiring other missing data methods such as imputation. Variables with greater than 50% missingness were excluded from analyses. Statistical significance was assessed at α=0.05 (two-tailed). Analyses were conducted in R version 4.3.1 using packages lme4 and glmmTMB (26, 27).

## Results

### Study population characteristics

From 69,932 initially eligible patients, 17,710 (25.3%) met all inclusion criteria and had activity at all required follow-up time points. The primary reason for exclusion was lack of documented activity at all follow-up time periods. The distribution of IMIDs was psoriasis (n=9,724), Crohn’s disease (n=4,235), ulcerative colitis (n=4,139), and psoriatic arthritis (n=3,656) (**Table 1**). Within each disease cohort, BMI distribution was 16-31% patients with HW, 27-35% patients with overweight, and 37-53% patients with obesity. Mean age ranged from 53.2 years in Crohn’s disease to 58.0 years in psoriasis. Female patients comprised 53-60% across disease cohorts. The study population was predominantly white race and non-Hispanic ethnicity (86-90%).

**Table 1:** Baseline characteristics of the population with psoriasis, psoriatic arthritis, ulcerative colitis, and Crohn’s disease.

Psoriasis					
		BMI Category			
Variable	Overall n = 9,724	18.5-24.9 n = 1,610	25-29.9 n = 2,945	30+ n = 5,169	p-value^1^
Age at index (years)	58.0 (12.6)	56.6 (14.2)	59.2 (12.6)	57.8 (12.0)	<0.001
Gender					<0.001
Female	5,168 (53%)	1,037 (64%)	1,348 (46%)	2,783 (54%)	
Male	4,546 (47%)	570 (35%)	1,597 (54%)	2,379 (46%)	
Race					<0.001
African American	400 (4%)	56 (3%)	100 (3%)	244 (5%)	
Asian	318 (3%)	107 (7%)	120 (4%)	91 (2%)	
Caucasian	8,632 (89%)	1,393 (87%)	2,613 (89%)	4,626 (89%)	
Ethnicity					<0.001
Hispanic	602 (6%)	67 (4%)	166 (6%)	369 (7%)	
Not Hispanic	8,867 (91%)	1,492 (93%)	2,685 (91%)	4,690 (91%)	
Elixhauser score					0.9
≤0	8,735 (90%)	1,452 (90%)	2,645 (90%)	4,638 (90%)	
>0	989 (10%)	158 (10%)	300 (10%)	531 (10%)	
Any high-risk diagnosis^2^	7,400 (76%)	907 (56%)	2,191 (74%)	4,302 (83%)	<0.001
5-ASA prescription	240 (2%)	38 (2%)	72 (2%)	130 (3%)	>0.9
TNF-α inhibitor prescription	1,527 (16%)	245 (15%)	479 (16%)	803 (16%)	0.6
Integrin inhibitor prescription	n<11	n<11	n<11	n<11	0.2
Interleukin inhibitor Prescription	1,044 (11%)	175 (11%)	258 (9%)	611 (12%)	<0.001
JAK inhibitor prescription	158 (2%)	24 (1%)	42 (1%)	92 (2%)	0.4
Corticosteroid prescription	2,338 (24%)	339 (21%)	652 (22%)	1,347 (26%)	<0.001
Number of biologics					0.041
0	7,330 (75%)	1,227 (76%)	2,261 (77%)	3,842 (74%)	
1+	2,384 (25%)	380 (24%)	684 (23%)	1,320 (26%)	
Psoriatic arthritis					
		BMI Category			
Variable	Overall n = 3,656	18.5-24.9 n = 595	25-29.9 n = 1,110	30+ n = 1,951	p-value^1^
Age at index (years)	57.9 (11.9)	57.0 (13.4)	58.8 (12.0)	57.6 (11.4)	0.003
Gender					<0.001
Female	2,010 (55%)	388 (65%)	511 (46%)	1,111 (57%)	
Male	1,640 (45%)	205 (34%)	599 (54%)	836 (43%)	
Race					<0.001
African American	101 (3%)	17 (3%)	21 (2%)	63 (3%)	
Asian	93 (3%)	30 (5%)	39 (4%)	24 (1%)	
Caucasian	3,318 (91%)	528 (89%)	1,004 (90%)	1,786 (92%)	
Ethnicity					0.002
Hispanic	198 (5%)	27 (5%)	45 (4%)	126 (6%)	
Not Hispanic	3,348 (92%)	545 (92%)	1,022 (92%)	1,781 (91%)	
Elixhauser score					0.4
≤0	3,239 (89%)	526 (88%)	973 (88%)	1,740 (89%)	
>0	417 (11%)	69 (12%)	137 (12%)	211 (11%)	
Any high-risk diagnosis^2^	2,568 (70%)	300 (50%)	746 (67%)	1,522 (78%)	<0.001
5-ASA prescription	176 (5%)	25 (4%)	52 (5%)	99 (5%)	0.7
TNF-α inhibitor prescription	1,169 (32%)	190 (32%)	373 (34%)	606 (31%)	0.3
Integrin inhibitor prescription					
Interleukin inhibitor Prescription	675 (18%)	110 (18%)	175 (16%)	390 (20%)	0.015
JAK inhibitor prescription	130 (4%)	18 (3%)	35 (3%)	77 (4%)	0.4
Corticosteroid prescription	1,092 (30%)	165 (28%)	296 (27%)	631 (32%)	0.002
Number of biologics					0.4
0	1,959 (54%)	327 (55%)	607 (55%)	1,025 (53%)	
1+	1,691 (46%)	266 (45%)	503 (45%)	922 (47%)	
Ulcerative colitis					
		BMI Category			
Variable	Overall n = 4,139	18.5-24.9 n = 1,140	25-29.9 n = 1,436	30+ n = 1,563	p-value^1^
Age at index (years)	55.6 (14.9)	50.9 (16.5)	57.1 (14.6)	57.8 (13.1)	<0.001
Gender					<0.001
Female	2,265 (55%)	754 (66%)	663 (46%)	848 (54%)	
Male	1,871 (45%)	384 (34%)	773 (54%)	714 (46%)	
Race					<0.001
African American	321 (8%)	48 (4%)	105 (7%)	***^3^	
Asian	107 (3%)	58 (5%)	40 (3%)	***^3^	
Caucasian	3,589 (87%)	996 (87%)	1,251 (87%)	1,342 (86%)	
Ethnicity					0.056
Hispanic	169 (4%)	43 (4%)	56 (4%)	70 (4%)	
Not Hispanic	3,854 (93%)	1,052 (92%)	1,342 (93%)	1,460 (93%)	
Elixhauser score					0.12
≤0	3,715 (90%)	1,041 (91%)	1,282 (89%)	1,392 (89%)	
>0	424 (10%)	99 (9%)	154 (11%)	171 (11%)	
Any high-risk diagnosis^2^	2,726 (66%)	504 (44%)	961 (67%)	1,261 (81%)	<0.001
5-ASA prescription	1,863 (45%)	553 (49%)	640 (45%)	670 (43%)	0.013
TNF-α inhibitor prescription	361 (9%)	113 (10%)	118 (8%)	130 (8%)	0.2
Integrin inhibitor prescription	64 (2%)	26 (2%)	23 (2%)	15 (1%)	0.022
Interleukin inhibitor Prescription	48 (1%)	20 (2%)	15 (1%)	13 (1%)	0.076
JAK inhibitor prescription	60 (1%)	20 (2%)	17 (1%)	23 (1%)	0.5
Corticosteroid prescription	1,177 (28%)	321 (28%)	373 (26%)	483 (31%)	0.011
Number of biologics					0.018
0	3,689 (89%)	991 (87%)	1,285 (89%)	1,413 (90%)	
1+	447 (11%)	147 (13%)	151 (11%)	149 (10%)	
Crohn’s disease					
		BMI Category			
Variable	Overall n = 4,235	18.5-24.9 n = 1,282	25-29.9 n = 1,412	30+ n = 1,541	p-value^1^
Age at index (years)	53.3 (15.4)	50.2 (17.0)	54.7 (15.5)	54.5 (13.5)	<0.001
Gender					<0.001
Female	2,543 (60%)	811 (63%)	732 (52%)	1,000 (65%)	
Male	1,689 (40%)	469 (37%)	680 (48%)	540 (35%)	
Race					<0.001
African American	314 (7%)	69 (5%)	89 (6%)	156 (10%)	
Asian	57 (1%)	30 (2%)	14 (1%)	13 (1%)	
Caucasian	3,760 (89%)	1,146 (89%)	1,278 (91%)	1,336 (87%)	
Ethnicity					0.002
Hispanic	139 (3%)	46 (4%)	51 (4%)	42 (3%)	
Not Hispanic	3,981 (94%)	1,185 (92%)	1,323 (94%)	1,473 (96%)	
Elixhauser score					0.3
≤0	3,806 (90%)	1,158 (90%)	1,277 (90%)	1,371 (89%)	
>0	429 (10%)	124 (10%)	135 (10%)	170 (11%)	
Any high-risk diagnosis^2^	2,590 (61%)	554 (43%)	866 (61%)	1,170 (76%)	<0.001
5-ASA prescription	879 (21%)	235 (18%)	303 (21%)	341 (22%)	0.034
TNF-α inhibitor prescription	737 (17%)	240 (19%)	230 (16%)	267 (17%)	0.2
Integrin inhibitor prescription	76 (2%)	36 (3%)	15 (1%)	25 (2%)	0.002
Interleukin inhibitor Prescription	265 (6%)	96 (7%)	85 (6%)	84 (5%)	0.076
JAK inhibitor prescription	20 (0%)	n<11	n<11	n<11	0.7
Corticosteroid prescription	1,405 (33%)	367 (29%)	448 (32%)	590 (38%)	<0.001
Number of biologics					0.007
0	3,214 (76%)	932 (73%)	1,096 (78%)	1,186 (77%)	
1+	1,018 (24%)	348 (27%)	316 (22%)	354 (23%)	

^1^ Kruskal-Wallis rank sum test for continuous variables; Pearson’s Chi-squared test for categorical variables; Fisher’s exact test for categorical variables with small cell counts. All statistical comparisons are among the 3 BMI categories.

^2^ Diagnoses include hypertension, dyslipidemia, type 2 diabetes, obstructive sleep apnea, and cardiovascular disease.

^3^ Information removed due to small sample size <11 in at least one cell.

Baseline characteristics varied significantly across BMI categories within each disease cohort. Patients with obesity demonstrated substantially higher prevalence of high-risk cardiovascular and metabolic conditions compared to patients with HW. Across all four diseases, the proportion of patients with at least one high-risk diagnosis (hypertension, dyslipidemia, type 2 diabetes, obstructive sleep apnea, or cardiovascular disease) ranged from 43-56% in patients with HW compared with 76-83% in patients with obesity (all p<0.001). Specific patterns included: psoriasis (83% vs 56%), psoriatic arthritis (78% vs 50%), ulcerative colitis (80% vs 44%), and Crohn’s disease (76% vs 43%).

Medication use at baseline showed modest variation across BMI categories. Biologic therapy prescriptions ranged from 11-46% depending on disease type, with psoriatic arthritis showing the highest use (46%) and ulcerative colitis the lowest (11%). Corticosteroid prescriptions were higher in patients with obesity across most disease cohorts (26-38%) compared to patients with HW (21-29%).

### C-reactive protein associations

**Figure 1:**
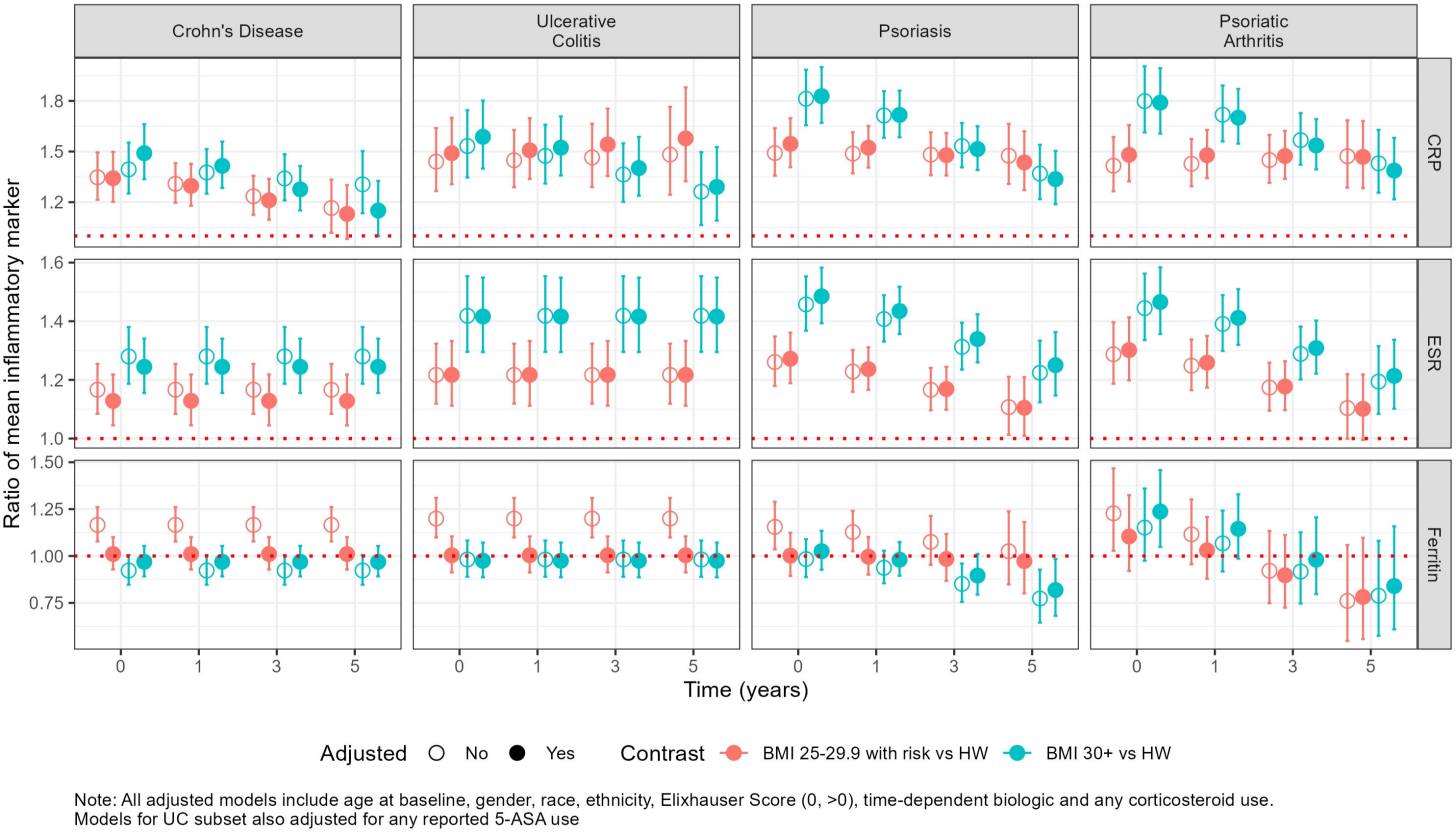
Ratio of means for BMI groups by diagnosis and inflammatory markers over time.

Patients with obesity demonstrated significantly elevated CRP levels compared with patients with HW across all four disease cohorts (**Figure 1**). At baseline, adjusted ratios of mean CRP for patients with BMI ≥30 kg/m² ranged from 49% to 83% higher across diseases, with the highest elevations observed in patients with psoriasis (83% higher, 95% CI: 67-100%) and patients with psoriatic arthritis (79% higher, 95% CI: 61-99%). Patients diagnosed with ulcerative colitis showed a baseline ratio of 59% higher (95% CI: 40-80%), while patients with Crohn’s disease demonstrated 49% higher CRP (95% CI: 34-66%). All associations were statistically significant (p<0.001).

Patients in the overweight with high-risk condition group also showed CRP elevations. At baseline, adjusted ratios for this group relative to the HW group ranged from 34% (95% CI: 20-50%) to 55% higher (95% CI: 41-70%) across the four diseases, with all estimates statistically significant (p<0.05). These associations attenuated over time but remained significant through year 5 in most cohorts.

### Erythrocyte sedimentation rate associations

ESR associations with obesity mirrored CRP patterns across all four conditions (**Figure 1**). Baseline adjusted ratios for patients with BMI ≥30 kg/m² ranged from 24% to 48% higher, with patients with psoriasis showing the highest elevation (48%, 95% CI: 39-58% higher). Patients with psoriatic arthritis, ulcerative colitis, and Crohn’s disease demonstrated ratios of 47% higher (95% CI: 36-58%), 42% higher (95% CI: 29-55%), and 24% higher (95% CI: 16-34%) ESR, respectively. All associations were statistically significant (p<0.001).

Overweight patients with high-risk conditions exhibited ESR elevations with baseline ratios between 13% (95% CI: 5-22%) and 30% higher (95% CI: 20-41%) across diseases (all p<0.001). Positive associations between BMI and ESR persisted over time with slight attenuation in psoriasis and psoriatic arthritis cohorts. Year 5 ratios ranged from 10% (95% CI: 0-22%) to 22% (95% CI: 11-33%) higher across diseases.

### Ferritin associations

In contrast to CRP and ESR, ferritin showed near null associations with obesity across all disease cohorts and time points (**Figure 1**). For patients with BMI ≥30 kg/m², adjusted ratios relative to HW ranged from 3% lower (95% CI: -11% - 5%) to 24% higher (95% CI: 5-46%) throughout the five-year follow-up period. No statistically significant associations were observed in Crohn’s disease or ulcerative colitis cohorts at any time point. Psoriasis and psoriatic arthritis showed similar near null patterns.

Patients with overweight plus high-risk conditions also showed no statistically significant ferritin elevations across any of the four conditions. Adjusted associations at baseline were near null for Crohn’s disease (1.01; 95% CI: 0.93-1.10), ulcerative colitis (1.00; 95% CI: 0.91-1.10), psoriasis (1.00; 95% CI: 0.89-1.12), and psoriatic arthritis (1.10; 95% CI: 0.92-1.32), with similar patterns persisting through follow-up.

## Discussion

This study demonstrated that obesity and overweight are associated with persistent elevation of CRP and ESR across four immunologically distinct IMIDs over five years of follow-up. While these associations attenuated over time, they remained statistically significant in most cohorts through year 5, supporting the hypothesis that adipose tissue may represent a sustained rather than transient inflammatory source. The consistent associations occurred across diseases with fundamentally different immunopathology. In contrast, ferritin showed near null associations with obesity, a pattern more likely attributable to co-regulation by iron stores than to selective activation of the inflammatory response. These findings are consistent with the possibility of a shared adipose tissue-associated inflammatory pattern that is not confined to a single immunopathological pathway, although the observational design supports association rather than causation.

Our findings are consistent with and expand on studies that have examined BMI-inflammatory marker relationships within individual IMIDs. In a study of patients with psoriasis, Reich et al. (19) documented elevated hs-CRP in patients with higher BMI using pooled clinical trial data, and Rodríguez-Cerdeira et al. reported similar elevations in inflammatory biomarkers among patients with co-occurring obesity and psoriasis (28). In a case-control study of patients with Crohn’s disease and healthy controls, Nic Suibhne et al. found that higher BMI associated with higher CRP (29), and Greuter et al. (20) demonstrated that obesity was an independent negative predictor of disease remission in CD but not in UC. In a study of patients with psoriatic arthritis and obesity, Klingberg et al. provided interventional evidence that intentional weight loss reduces CRP and improves disease activity scores, supporting the premise that the associations observed in our study may be modifiable (30).

Prior studies have examined ESR in relation to BMI far less frequently than CRP within these specific patient populations. Data on the BMI-ferritin relationship in IMIDs are also lacking. Furthermore, existing evidence comes predominantly from single-disease, cross-sectional designs. To our knowledge, no study has simultaneously compared BMI-inflammatory marker associations across multiple IMIDs within a unified longitudinal study design. Our design addresses this gap and provides evidence that the CRP and ESR elevations associated with obesity may not be disease-specific phenomena but instead may reflect a shared pattern of adiposity-driven inflammation that persists over multiple years.

### Mechanistic interpretation

The observed patterns support a model in which adipose tissue functions as an immunologically active organ, contributing systemic inflammation independent of disease-specific immunopathology. In obesity, stressed adipocytes trigger macrophage recruitment and M1 polarization, establishing a pro-inflammatory microenvironment with elevated IL-6, TNF-α, and IL-1β production (31, 32). IL-6 serves as the primary driver of hepatic acute-phase response, directly stimulating CRP synthesis (33). ESR elevation reflects increased fibrinogen and immunoglobulin concentrations, both IL-6-regulated (34).

The cross-disease consistency of CRP and ESR elevations suggest the possibility of adipose-derived inflammation operating across diverse disease contexts rather than being confined to a single immunopathological pathway. Despite fundamental differences in disease pathophysiology, i.e., intestinal dysfunction and dysbiosis in IBD (35) versus IL-17/IL-23 axis activation in psoriatic diseases (36), obesity produced similar CRP and ESR associations across all four conditions. This uniformity is consistent with parallel operation of adipose and disease-specific inflammatory pathways, though the absence of a non-IMID comparator group means that interactive effects between adiposity and disease-specific immune activation cannot be excluded. The somewhat larger CRP and ESR elevations observed in psoriasis and psoriatic arthritis compared with IBD may reflect the systemic, multisystem nature of psoriatic disease. Whereas IBD inflammation is anatomically concentrated in the gut, psoriatic disease involves the skin, joints, entheses, and vasculature and is accompanied by a well-described burden of cardiometabolic comorbidity (37), so a larger fraction of the circulating acute-phase response may be generated outside any single organ. Adipose-derived inflammation may therefore disperse more readily with the diffuse inflammatory load of psoriatic disease than with the more compartmentalized inflammation of IBD, although this interpretation is hypothesis-generating and cannot be confirmed without direct measures of disease activity.

The divergent pattern in ferritin associations compared with CRP and ESR most likely reflects ferritin’s distinct regulatory biology rather than selective adipose-derived inflammation. Unlike CRP and ESR, which are primarily IL-6-regulated, ferritin serves dual functions as both acute-phase reactant and iron storage protein, with transcriptional control through iron-responsive elements (7). Ferritin is also co-regulated by iron status, and although it rises as an acute-phase reactant, its response to IL-6 is more variable and is modulated by hepcidin-mediated iron handling (38, 39). Notably, obesity is frequently associated with hyperferritinemia despite low circulating iron (40). A near-null BMI-ferritin association in our cohort more likely reflects the competing influences of inflammation and iron metabolism on ferritin versus an absence of adipose-driven inflammation. The contrast between ferritin and the IL-6-driven markers likely reflects a difference in marker regulation rather than in the scope of adipose inflammation.

### Temporal patterns

A key finding of this study is that CRP and ESR elevations persisted across the five-year follow-up period in the higher BMI categories, though with gradual attenuation over time. At baseline, patients with obesity demonstrated CRP levels 49–83% higher than HW counterparts; by year 5, these elevations had decreased but remained statistically significant in most cohorts. This temporal pattern is consistent with the hypothesis that adipose tissue may function as a sustained, rather than transient, source of systemic inflammation.

### Clinical implications

These findings represent a preliminary step towards understanding the clinical implications of inflammatory marker interpretations in patients with IMIDs. Clinicians, including specialists who treat these IMIDs, e.g., gastroenterologists, rheumatologists, or dermatologists, may benefit from considering BMI when assessing CRP and ESR values, recognizing that elevations in patients with obesity may partially reflect adipose-driven inflammation rather than disease activity alone. For example, a CRP value of 10 mg/L might represent moderate disease activity in a patient with HW but could occur from adiposity-driven inflammation in a patient with obesity and well-controlled disease. This distinction becomes particularly relevant when making treatment decisions based on inflammatory markers, such as escalating immunosuppression or initiating biologic therapy.

The persistence of elevated inflammatory markers over five years supports further exploration of long-term weight management as a therapeutic strategy for patients with IMIDs. Current FDA criteria for pharmacologic weight management with GLP-1 receptor agonists include a BMI in the overweight range (25.0-29.9 kg/m²) with at least one high-risk comorbidity, or a BMI in the obesity range. A substantial proportion of patients with IMIDs in our cohort met these thresholds and also demonstrated elevated CRP and ESR, suggesting that these individuals may stand to benefit from targeted weight management interventions.

### Strengths and limitations

This study’s primary strength is its multi-disease design examining four immunologically distinct conditions simultaneously, enabling assessment of consistency versus heterogeneity of inflammatory patterns across IMIDs. The large sample size (n=17,710) with longitudinal follow-up over five years provides robust evidence for temporal persistence of associations. Inclusion of multiple inflammatory markers enabled specificity assessment. Model adjustments including marginal structural models with inverse probability weighting addressed potential confounding from time-varying factors. Use of real-world data from integrated EHR and claims provides generalizable evidence from routine clinical practice.

Several limitations warrant consideration. The requirement for documented activity at every follow-up window resulted in sample size reduction, with only 25.3% of initially eligible patients retained. This attrition may have introduced selection bias. Patients with complete follow-up likely differ systematically from those excluded; they may be more engaged with the health system, have more active or closely monitored disease, or carry a greater comorbidity burden prompting regular visits. Because these factors are also associated with both BMI and inflammatory marker levels, this study may not generalize to patients who engage with the healthcare system less frequently.

BMI does not distinguish lean muscle mass from adipose tissue or capture fat distribution patterns, particularly visceral versus subcutaneous adiposity, which may have distinct inflammatory implications. Relatedly, the “overweight with a high-risk condition” group was defined to mirror pharmacologic weight-management eligibility rather than a biological threshold, and the presence of cardiometabolic comorbidities (hypertension, diabetes, dyslipidemia, cardiovascular disease) means elevated inflammatory markers in this group may partly reflect those comorbidities and their treatment rather than adiposity per se. Estimates for this group should therefore be read as describing a clinically actionable population rather than an isolated effect of overweight.

Inflammatory markers were not obtained systematically at standardized time points, and laboratory assay methods may vary across contributing healthcare organizations. Test ordering also reflected clinical practice rather than protocol, which is especially relevant for ferritin. Ferritin is ordered far more routinely in IBD (where iron-deficiency anemia is monitored) than in psoriatic disease, so ferritin availability and the clinical context prompting the test differ systematically across cohorts. This differential ascertainment may influence the ferritin findings and limits direct comparison of ferritin associations between IBD and psoriatic cohorts.

The observational design precludes causal inference; we cannot determine whether elevated markers result from adipose-driven inflammation, more severe underlying disease in patients with obesity, or bidirectional relationships.

Residual confounding by disease severity is a particular concern: we lacked direct measures of disease activity (e.g., endoscopic or histologic scores in IBD, PASI or joint counts in psoriatic disease), and patients with obesity may have more active underlying disease that independently elevates inflammatory markers. Although our models adjusted for biologic and corticosteroid use as proxies for disease severity, these do not fully capture the construct, so some of the observed association may reflect unmeasured severity rather than adiposity itself. Unmeasured confounding from diet quality, physical activity, smoking status, and disease phenotype remains possible despite comprehensive adjustment.

Generalizability is limited to adults receiving care from US healthcare systems, and findings may not extend to uninsured populations, pediatric patients, or international settings. Without a non-IMID comparator group, we cannot definitively determine whether the observed BMI-inflammatory marker associations operate independently of disease status. Although BMI-CRP associations are well-documented in general populations without IMIDs (41, 42), inclusion of such a comparator group would have enabled direct assessment of whether the magnitude of associations differs by disease status.

## Conclusion

Obesity is associated with persistent CRP and ESR elevations across Crohn’s disease, ulcerative colitis, psoriasis, and psoriatic arthritis, with consistency across immunologically distinct diseases suggesting the possibility of a shared adipose tissue-driven inflammatory mechanism. Ferritin’s near null association with BMI most likely reflects its distinct, iron--coupled regulation rather than indicating that adipose-driven inflammation is selective or absent. These findings support BMI-informed interpretation of inflammatory markers for patients with IMIDs and highlight weight management as a potentially underutilized therapeutic strategy for treating these IMIDs. Future studies should further explore biologic mechanisms that could cause different observed inflammatory markers across BMI categories in patients with IMIDs. Future interventional studies should also assess whether weight reduction through behavioral, pharmacologic, or surgical approaches modulates inflammatory markers and improves disease outcomes across these conditions. Such studies could address whether the observed associations reflect modifiable inflammatory burden, thereby informing evidence-based integration of weight management into comprehensive IMID treatment algorithms.

## Data Availability

The data analyzed in this study is subject to the following licenses/restrictions: data from the Optum Labs Data Warehouse are proprietary and not publicly available; researchers interested in replicating or extending these analyses would need to obtain independent access through Optum Labs. Requests to access these datasets should be directed to sshields@amga.org.
