# Supplementary material for: Inflammatory marker profiles vary by BMI across Crohn’s disease, ulcerative colitis, psoriasis, and psoriatic arthritis
Source: Front Endocrinol (Lausanne). 2026 Jul 17;17:1851905. doi: 10.3389/fendo.2026.1851905 (PMC13423644; doi:10.3389/fendo.2026.1851905)
Supplement: Supplementary file 1 [file SupplementaryFile1.docx]

Supplementary Material

Supplementary Table 1: List of diagnosis codes used to identify immune-mediated inflammatory diseases

| **Condition** | **Code System** | **Code** |
| --- | --- | --- |
| Crohn’s disease | ICD10 | K50.* |
| Crohn’s disease | ICD9 | 555.* |
| Ulcerative colitis | ICD10 | K51.* |
| Ulcerative colitis | ICD9 | 556.* |
| Psoriasis | ICD10 | L40.* |
| Psoriasis | ICD9 | 696.1 |
| Psoriatic arthritis | ICD10 | L40.5* |
| Psoriatic arthritis | ICD9 | 696 |


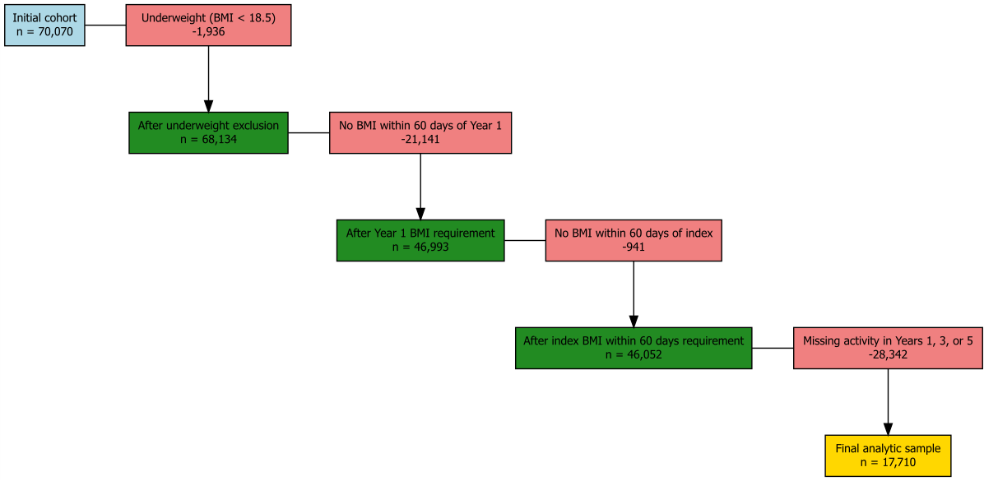
Supplementary Table 2: Patient attrition table

Supplementary Table 3: Ratio of inflammatory marker outcomes by BMI groups, adjusted status^1^, and time.

|  | | | **Crohn’s Disease** | | **Psoriatic Arthritis** | | **Psoriasis** | | **Ulcerative Colitis** | |
| --- | --- | --- | --- | --- | --- | --- | --- | --- | --- | --- |
| **Marker** | **Comparison** | **Time (years)** | **No** | **Yes** | **No** | **Yes** | **No** | **Yes** | **No** | **Yes** |
| CRP | BMI 25-29.9 with risk vs HW | 0 | 1.35 (1.21, 1.50) | 1.34 (1.20, 1.50) | 1.42 (1.26, 1.59) | 1.48 (1.32, 1.66) | 1.49 (1.36, 1.64) | 1.55 (1.41, 1.70) | 1.44 (1.27, 1.64) | 1.49 (1.31, 1.70) |
|  |  | 1 | 1.31 (1.20, 1.43) | 1.30 (1.18, 1.43) | 1.43 (1.29, 1.57) | 1.48 (1.34, 1.63) | 1.49 (1.37, 1.61) | 1.52 (1.40, 1.65) | 1.45 (1.29, 1.63) | 1.51 (1.34, 1.70) |
|  |  | 3 | 1.24 (1.12, 1.36) | 1.21 (1.10, 1.34) | 1.45 (1.31, 1.60) | 1.47 (1.34, 1.62) | 1.48 (1.36, 1.61) | 1.48 (1.36, 1.61) | 1.47 (1.29, 1.66) | 1.54 (1.36, 1.75) |
|  |  | 5 | 1.17 (1.02, 1.33) | 1.13 (0.98, 1.30) | 1.47 (1.29, 1.69) | 1.47 (1.28, 1.68) | 1.48 (1.31, 1.66) | 1.44 (1.27, 1.62) | 1.48 (1.24, 1.77) | 1.58 (1.32, 1.88) |
| ESR |  | 0 | 1.17 (1.08, 1.25) | 1.13 (1.05, 1.22) | 1.29 (1.19, 1.40) | 1.30 (1.20, 1.41) | 1.26 (1.18, 1.35) | 1.27 (1.19, 1.36) | 1.22 (1.12, 1.32) | 1.22 (1.11, 1.33) |
|  |  | 1 | 1.17 (1.08, 1.25) | 1.13 (1.05, 1.22) | 1.25 (1.16, 1.34) | 1.26 (1.17, 1.35) | 1.23 (1.16, 1.30) | 1.24 (1.17, 1.31) | 1.22 (1.12, 1.32) | 1.22 (1.11, 1.33) |
|  |  | 3 | 1.17 (1.08, 1.25) | 1.13 (1.05, 1.22) | 1.17 (1.09, 1.26) | 1.18 (1.10, 1.26) | 1.17 (1.10, 1.24) | 1.17 (1.10, 1.24) | 1.22 (1.12, 1.32) | 1.22 (1.11, 1.33) |
|  |  | 5 | 1.17 (1.08, 1.25) | 1.13 (1.05, 1.22) | 1.10 (1.00, 1.22) | 1.10 (1.00, 1.22) | 1.11 (1.01, 1.21) | 1.10 (1.01, 1.21) | 1.22 (1.12, 1.32) | 1.22 (1.11, 1.33) |
| Ferritin |  | 0 | 1.17 (1.08, 1.26) | 1.01 (0.93, 1.10) | 1.23 (1.03, 1.47) | 1.10 (0.92, 1.32) | 1.16 (1.04, 1.29) | 1.00 (0.89, 1.12) | 1.20 (1.10, 1.31) | 1.00 (0.91, 1.10) |
|  |  | 1 | 1.17 (1.08, 1.26) | 1.01 (0.93, 1.10) | 1.12 (0.96, 1.30) | 1.03 (0.88, 1.21) | 1.13 (1.03, 1.24) | 1.00 (0.90, 1.10) | 1.20 (1.10, 1.31) | 1.00 (0.91, 1.10) |
|  |  | 3 | 1.17 (1.08, 1.26) | 1.01 (0.93, 1.10) | 0.92 (0.75, 1.13) | 0.90 (0.72, 1.11) | 1.08 (0.95, 1.21) | 0.98 (0.87, 1.12) | 1.20 (1.10, 1.31) | 1.00 (0.91, 1.10) |
|  |  | 5 | 1.17 (1.08, 1.26) | 1.01 (0.93, 1.10) | 0.76 (0.55, 1.06) | 0.78 (0.56, 1.10) | 1.03 (0.85, 1.24) | 0.97 (0.80, 1.18) | 1.20 (1.10, 1.31) | 1.00 (0.91, 1.10) |
| CRP | BMI 30+ vs HW | 0 | 1.39 (1.25, 1.55) | 1.49 (1.34, 1.66) | 1.80 (1.61, 2.01) | 1.79 (1.61, 1.99) | 1.81 (1.66, 1.98) | 1.83 (1.67, 2.00) | 1.53 (1.35, 1.75) | 1.59 (1.40, 1.80) |
|  |  | 1 | 1.38 (1.25, 1.51) | 1.42 (1.28, 1.56) | 1.72 (1.56, 1.89) | 1.70 (1.55, 1.87) | 1.71 (1.58, 1.86) | 1.72 (1.58, 1.86) | 1.47 (1.31, 1.66) | 1.52 (1.36, 1.71) |
|  |  | 3 | 1.34 (1.21, 1.48) | 1.28 (1.15, 1.41) | 1.57 (1.42, 1.73) | 1.54 (1.39, 1.69) | 1.53 (1.41, 1.67) | 1.52 (1.39, 1.65) | 1.36 (1.20, 1.55) | 1.40 (1.24, 1.59) |
|  |  | 5 | 1.31 (1.13, 1.50) | 1.15 (1.00, 1.33) | 1.43 (1.26, 1.63) | 1.39 (1.22, 1.58) | 1.37 (1.22, 1.54) | 1.34 (1.19, 1.50) | 1.26 (1.06, 1.50) | 1.29 (1.09, 1.53) |
| ESR |  | 0 | 1.28 (1.19, 1.38) | 1.24 (1.16, 1.34) | 1.44 (1.34, 1.56) | 1.47 (1.36, 1.58) | 1.46 (1.37, 1.55) | 1.48 (1.39, 1.58) | 1.42 (1.30, 1.55) | 1.42 (1.29, 1.55) |
|  |  | 1 | 1.28 (1.19, 1.38) | 1.24 (1.16, 1.34) | 1.39 (1.30, 1.49) | 1.41 (1.32, 1.51) | 1.41 (1.33, 1.49) | 1.43 (1.36, 1.52) | 1.42 (1.30, 1.55) | 1.42 (1.29, 1.55) |
|  |  | 3 | 1.28 (1.19, 1.38) | 1.24 (1.16, 1.34) | 1.29 (1.20, 1.38) | 1.31 (1.22, 1.40) | 1.31 (1.24, 1.39) | 1.34 (1.26, 1.42) | 1.42 (1.30, 1.55) | 1.42 (1.29, 1.55) |
|  |  | 5 | 1.28 (1.19, 1.38) | 1.24 (1.16, 1.34) | 1.19 (1.08, 1.32) | 1.21 (1.10, 1.34) | 1.22 (1.12, 1.33) | 1.25 (1.15, 1.36) | 1.42 (1.30, 1.55) | 1.42 (1.29, 1.55) |
| Ferritin |  | 0 | 0.92 (0.85, 1.01) | 0.97 (0.89, 1.05) | 1.15 (0.97, 1.36) | 1.24 (1.05, 1.46) | 0.98 (0.89, 1.09) | 1.03 (0.93, 1.13) | 0.98 (0.89, 1.08) | 0.97 (0.89, 1.07) |
|  |  | 1 | 0.92 (0.85, 1.01) | 0.97 (0.89, 1.05) | 1.07 (0.92, 1.24) | 1.14 (0.99, 1.33) | 0.94 (0.85, 1.03) | 0.98 (0.89, 1.07) | 0.98 (0.89, 1.08) | 0.97 (0.89, 1.07) |
|  |  | 3 | 0.92 (0.85, 1.01) | 0.97 (0.89, 1.05) | 0.92 (0.75, 1.13) | 0.98 (0.80, 1.21) | 0.85 (0.76, 0.96) | 0.90 (0.79, 1.01) | 0.98 (0.89, 1.08) | 0.97 (0.89, 1.07) |
|  |  | 5 | 0.92 (0.85, 1.01) | 0.97 (0.89, 1.05) | 0.79 (0.57, 1.08) | 0.84 (0.61, 1.16) | 0.77 (0.64, 0.93) | 0.82 (0.68, 0.98) | 0.98 (0.89, 1.08) | 0.97 (0.89, 1.07) |

# ^1^All adjusted models include age at baseline, gender, race, ethnicity, Elixhause Score (0,>0), time-dependent biologic, and any corticosteroid use. Models for UC diagnoses also adjusted for any 5-ASA use.
